# Preclinical Investigation in Neuroprotective Effects of the GPR55 Ligand VCE-006.1 in Experimental Models of Parkinson’s Disease and Amyotrophic Lateral Sclerosis

**DOI:** 10.3390/molecules26247643

**Published:** 2021-12-16

**Authors:** Sonia Burgaz, Concepción García, Claudia Gonzalo-Consuegra, Marta Gómez-Almería, Francisco Ruiz-Pino, Juan Diego Unciti, María Gómez-Cañas, Juan Alcalde, Paula Morales, Nadine Jagerovic, Carmen Rodríguez-Cueto, Eva de Lago, Eduardo Muñoz, Javier Fernández-Ruiz

**Affiliations:** 1 Instituto Universitario de Investigación en Neuroquímica, Departamento de Bioquímica y Biología Molecular, Facultad de Medicina, Universidad Complutense, 28040 Madrid, Spain; soniabur@ucm.es (S.B.); conchig@med.ucm.es (C.G.); clagon11@ucm.es (C.G.-C.); margom27@ucm.es (M.G.-A.); mgc@med.ucm.es (M.G.-C.); jualcald@ucm.es (J.A.); carc@med.ucm.es (C.R.-C.); elagofem@med.ucm.es (E.d.L.); 2 Centro de Investigación Biomédica en Red de Enfermedades Neurodegenerativas (CIBERNED), 28040 Madrid, Spain; 3 Instituto Ramón y Cajal de Investigación Sanitaria (IRYCIS), 28040 Madrid, Spain; 4 Emerald Health Biotechnology España, 14014 Córdoba, Spain; b62rupif@uco.es (F.R.-P.); jdunciti@gmail.com (J.D.U.); fi1muble@uco.es (E.M.); 5 Instituto de Química Médica, CSIC, 28006 Madrid, Spain; paula.morales@iqm.csic.es (P.M.); nadine@iqm.csic.es (N.J.); 6 Instituto Maimónides de Investigación Biomédica de Córdoba (IMIBIC), 14004 Córdoba, Spain; 7 Department of Cellular Biology, Physiology and Immunology, University of Córdoba, 14071 Córdoba, Spain; 8 Hospital Universitario Reina Sofía, 14004 Córdoba, Spain

**Keywords:** cannabinoids, GPR55 receptors, VCE-006.1, chromenopyrazole, Parkinson’s disease, 6-hydroxydopamine, lipopolysaccharide, amyotrophic lateral sclerosis, mSOD1 mice, TDP-43 transgenic mice

## Abstract

Cannabinoids act as pleiotropic compounds exerting, among others, a broad-spectrum of neuroprotective effects. These effects have been investigated in the last years in different preclinical models of neurodegeneration, with the cannabinoid type-1 (CB_1_) and type-2 (CB_2_) receptors concentrating an important part of this research. However, the issue has also been extended to additional targets that are also active for cannabinoids, such as the orphan G-protein receptor 55 (GPR55). In the present study, we investigated the neuroprotective potential of VCE-006.1, a chromenopyrazole derivative with biased orthosteric and positive allosteric modulator activity at GPR55, in murine models of two neurodegenerative diseases. First, we proved that VCE-006.1 alone could induce ERK1/2 activation and calcium mobilization, as well as increase cAMP response but only in the presence of lysophosphatidyl inositol. Next, we investigated this compound administered chronically in two neurotoxin-based models of Parkinson’s disease (PD), as well as in some cell-based models. VCE-006.1 was active in reversing the motor defects caused by 6-hydroxydopamine (6-OHDA) in the pole and the cylinder rearing tests, as well as the losses in tyrosine hydroxylase-containing neurons and the elevated glial reactivity detected in the substantia nigra. Similar cytoprotective effects were found in vitro in SH-SY5Y cells exposed to 6-OHDA. We also investigated VCE-006.1 in LPS-lesioned mice with similar beneficial effects, except against glial reactivity and associated inflammatory events, which remained unaltered, a fact confirmed in BV2 cells treated with LPS and VCE-006.1. We also analyzed GPR55 in these in vivo models with no changes in its gene expression, although GPR55 was down-regulated in BV2 cells treated with LPS, which may explain the lack of efficacy of VCE-006.1 in such an assay. Furthermore, we investigated VCE-006.1 in two genetic models of amyotrophic lateral sclerosis (ALS), mutant SOD1, or TDP-43 transgenic mice. Neither the neurological decline nor the deteriorated rotarod performance were prevented with this compound, and the same happened with the elevated microglial and astroglial reactivities, albeit modest spinal motor neuron preservation was achieved in both models. We also analyzed GPR55 in these in vivo models and found no changes in both TDP-43 transgenic and mSOD1 mice. Therefore, our findings support the view that targeting the GPR55 may afford neuroprotection in experimental PD, but not in ALS, thus stressing the specificities for the development of cannabinoid-based therapies in the different neurodegenerative disorders.

## 1. Introduction

Phytocannabinoids, the active constituents of the *Cannabis* plant, as well as endocannabinoids and synthetic cannabinoids, have been proposed as promising neuroprotective agents in accidental brain damage (e.g., stroke, brain trauma, spinal injury) and in chronic progressive disorders (e.g., Alzheimer’s disease, amyotrophic lateral sclerosis (ALS), Parkinson’s disease (PD), Huntington’s disease, and others) [1,2,3]. This potential derives from their pleiotropism and ability to activate numerous cytoprotective targets within the endocannabinoid system, but also outside this signaling system [3]. An important part of these neuroprotective properties described for cannabinoids have been related to the activation of the type-1 cannabinoid (CB_1_) receptor [1,2]. This receptor is predominantly located in neurons in the CNS, which facilitates its role in the control of excitotoxic damage in glutamatergic synapses [4], as well as a possible contribution in the autophagy-mediated elimination of protein aggregates [5]. Data supporting CB_1_ receptor-mediated neuroprotective effects have been collected in experimental models of Alzheimer’s disease [6,7,8], PD [9,10], ALS [11,12,13], Huntington’s disease [4,14,15,16], and multiple sclerosis [17,18].

Important neuroprotective effects have also been described for the activation of the type-2 cannabinoid (CB_2_) receptor [1,2,3,19]. This receptor is predominantly located in activated astrocytes and reactive microglial cells in the CNS of neuroinflammatory/neurodegenerative conditions, in which it becomes significantly up-regulated with the purpose to control glial toxicity for neurons as well as other beneficial effects [1,19]. Data supporting CB_2_ receptor-mediated neuroprotective effects have been collected in experimental models of Alzheimer’s disease and related dementias [7,20,21,22,23], PD [7,24,25,26,27], ALS [28,29,30,31,32], Huntington’s disease [33,34,35], and multiple sclerosis [36,37,38].

These broadly-demonstrated neuroprotective effects of cannabinoids have also been extended to additional targets, within or outside the endocannabinoid system, which are also active for cannabinoids [3]. This includes, for example, the nuclear receptors of the peroxisome proliferator-activating receptor (PPAR) family, which have been investigated for their role in the control of inflammatory/neurodegenerative events [39,40] in experimental PD [41,42,43,44], and, to a lower extent, in experimental ALS [45] and Alzheimer’s disease [46,47]. More recent data have indicated the orphan G-protein receptor 55 (GPR55) as an additional neuroprotective and anti-inflammatory target [48,49,50]. This has been investigated mainly in PD given the abundant presence of GPR55 receptors in the basal ganglia [51,52] and the important motor impairment found in mice lacking GPR55 [53].

GPR55 receptor was considered for years as an orphan receptor, but some recent evidence has positioned this receptor as a possible new cannabinoid receptor type [54]. However, such an assumption has been controversial due to the important differences in homology, conformational structure, pharmacology, signaling, and functional relevance shown by GPR55 compared to classic CB_1_ and CB_2_ receptors [55,56,57]. The human GPR55 protein has 319 amino acids and is also a member of the rhodopsin-like 7TM/GPCR family [55,57]. It was isolated and cloned in 1999, when it was found to be located in chromosome 2 (2q37) in humans [58]. Its naturally-occurring ligand is lysophosphatidyl inositol (LPI) [59]. Its pharmacology is complex and still remains to be clarified, including some non-cannabinoid compounds that do not bind CB_1_/CB_2_ receptors (e.g., GSK-494,581, CID-16020046 [60]), but also certain phytocannabinoids (e.g., cannabidiol), endocannabinoids (e.g., anandamide, 2-arachidonoylglycerol) and synthetic cannabinoids (e.g., WIN 55,212-2, HU-210, SR141716, AM251, methanandamide), which may also be active at other cannabinoid receptors [61,62]. GPR55 is widely distributed in the CNS, in particular in the basal ganglia, hippocampus, thalamus, and cerebellum [63], and is also present in the periphery (e.g., vasculature, gastrointestinal tract, bones, lung, spleen, liver, kidney, uterus) [64]. This distribution has prompted research on this receptor in relation to pathogenesis and/or development of novel therapies against different central and peripheral pathologies, including, as mentioned above, neurodegenerative disorders for which targeting GPR55 has been proposed as a promising anti-inflammatory and neuroprotective strategy [48,49,50,51].

In the present study, we have further investigated the neuroprotective potential of this new target for cannabinoids, using VCE-006.1, a chromenopyrazole derivative designed, synthesized, and investigated as GPR55 ligand in a previous study of our group [65]. VCE-006.1 is the compound 2-[2-(4-cyclohexylcarbonylpiperazinyl)ethyl]-2,4-dihydro-7-methoxy-4,4-dimethylchromeno[4,3-c]pyrazole (compound 23 in [65]), which showed affinity at the GPR55 receptor analyzed in a label-free cell-impedance-based assay in hGPR55-HEK293 cells, whereas having negligible or poor affinity for the CB_1_ and CB_2_ receptor (as measured in competitive radioligand assays), respectively [65]. The patent generated with this and other similar compounds [66] was acquired by the company Emerald Health Biotechnology-Spain in 2018, and the compound was renamed as VCE-006.1. In this study, we have extended the analysis of its activity at the GPR55 receptor, using several cell-based assays, which has situated this compound as a potential biased positive allosteric modulator (PAM) for the GPR55 receptor. Next, we have investigated its neuroprotective profile in vitro (cell-based assays) and in vivo (neurotoxin-based models or genetically-modified mice) models of two neurodegenerative diseases, PD and ALS, in which the potential of GPR55 as a neuroprotective target has been claimed [32,51].

## 2. Results

### 2.1. Studies on PAM Activity of VCE-006.1

**Figure 1:**
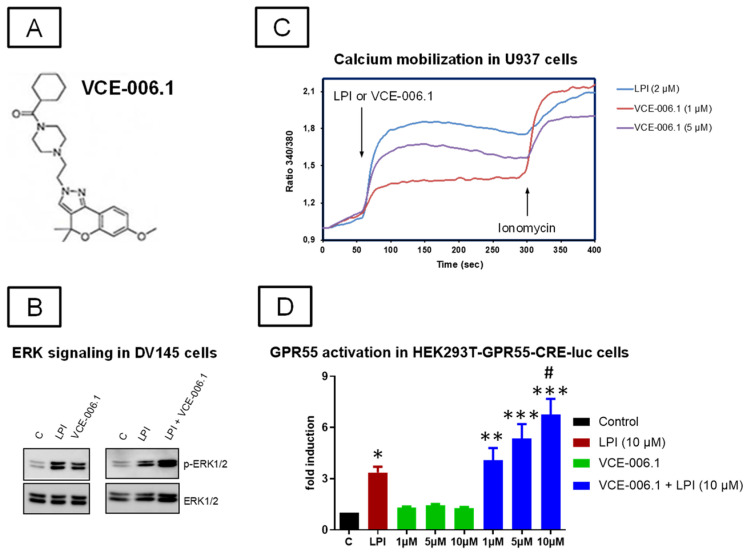
(**A**) Chemical structure of VCE-006.1. (**B**) VCE-006.1 and LPI induces ERK1/2 activation in DU145 cells. The cells were stimulated as indicated and the expression of phospho-ERK1/2 and total ERK1/2 determined by immunoblots. (**C**) VCE-006.1 and LPI induces [Ca^2+^] immobilization in U937 cells. U937 cells were loaded with Indo1-AM, treated with the compounds, and the calcium mobilization was measured by ratiometric fluorescence as indicated under Materials and Methods. (**D**) GPR55 activity of VCE-006.1 at different concentrations (1, 5, and 10 µM) in the absence or the presence of 10 µM LPI on HEK293T-GPR55-CRE-luc cells. Results are expressed as the fold induction of GPR55 activity and represent means ± SEM of data generated in 6 independent experiments, each conducted in triplicates. Statistical significance was determined by one-way ANOVA followed by the Tukey test (* *p* < 0.05, ** *p* < 0.01, *** *p* < 0.005 vs. control (basal) and VCE-006.1 alone; ^#^ *p* < 0.05 vs. LPI and VCE-006.1 (1 µM) + LPI).

Our first objective was to further explore the activity of VCE-006.1 (see chemical structure in Figure 1A) at the GPR55. Previous studies [65] have indicated VCE-006.1 to be a selective ligand of this receptor with activity as a partial agonist and having no relevant affinity at the classic CB_1_ and CB_2_ receptors tested in competitive radioligand binding assays. Here, we have explored canonical GPR55 signalling pathways in cells expressing the native receptor (DU145 and U937 cells) and in cells overexpressing the receptor (HEK-293-GPR55 cells). We found that both LPI and VCE-006.1 induced ERK1/2 phosphorylation in DU145 cells and that a combination of both further increased this phosphorylation (Figure 1B). Ca^2+^ mobilization in response to VCE-006.1 and LPI was studied in U937 cells, and as depicted in Figure 1C, both compounds were able to induce Ca^2+^ mobilization, with LPI being more potent than VCE-006.1, suggesting a different mode of action for each compound. Next, we stimulated HEK293-GPR55-CRE-Luc cells with either VCE-006.1 or LPI, separately or in combination, and the luciferase activity was measured as indicative of cAMP induction. VCE-006.1 did not induce CRE-Luc activity but significantly enhanced the effect of LPI as a potential orthosteric ligand (F(7,40) = 17.36, *p* < 0.0001; Figure 1D). Altogether, our results showed that VCE-006.1 activated GPR55 in a biased manner compared to LPI, showing characteristics of both partial orthosteric agonist and PAM depending on the specific cell assay used.

### 2.2. Studies in Experimental PD

**Figure 2:**
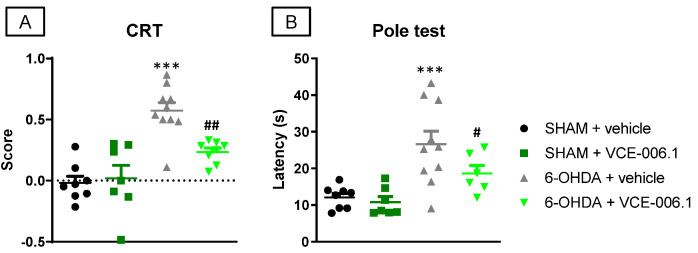
Response in the cylinder rearing test (**A**) and in the pole test (**B**) of male mice subjected to unilateral 6-OHDA lesions or sham-operated and daily treated with VCE-006.1 (20 mg/kg, i.p.) for 2 weeks. Values are means ± SEM of more than 6 animals per group. Data were assessed by one-way ANOVA followed by the Tukey test (*** *p* < 0.005 vs. the two sham-operated groups; ^#^ *p* < 0.05, ^##^ *p* < 0.01 vs. the vehicle-treated 6-OHDA lesioned mice).

Our second objective was to investigate this compound when administered chronically in two neurotoxin-based models of PD, as well as in some cell-based models of this disease. We first used a classic PD model of mitochondrial damage, 6-OHDA-lesioned mice, which proved the expected hemiparesis in the cylinder rearing test (Figure 2A) and an elevated latency to descend in the pole test (Figure 2B). VCE-006.1 was active in reversing these motor defects caused by 6-OHDA in the cylinder rearing test (F(3,29) = 17.49, *p* < 0.0001; Figure 2A) and in the pole test (F(3,27) = 8.803, *p* < 0.0005; Figure 2B), effects evident in 6-OHDA-lesioned mice, but absent in sham-operated mice.

**Figure 3:**
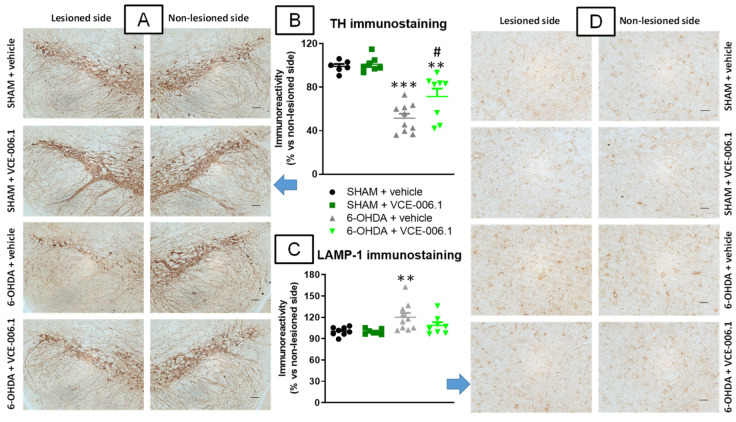
Quantification of TH (**B**) and LAMP-1 (**C**) immunoreactivities, including representative images (**A**) (TH; scale bar = 100 µm) and (**D**) (LAMP-1; scale bar = 50 µm)), measured in a selected area of the substantia nigra pars compacta of male mice subjected to unilateral 6-OHDA lesions or sham-operated and daily treated with VCE-006.1 (20 mg/kg, i.p.) for 2 weeks. Values correspond to % of the ipsilateral lesioned side vs. contralateral non-lesioned side and are expressed as means ± SEM of more than 6 animals per group. Data were assessed by one-way ANOVA followed by the Tukey test (** *p* < 0.01, *** *p* < 0.005 vs. the two sham-operated groups; ^#^ *p* < 0.05 vs. the vehicle-treated 6-OHDA lesioned mice).

These benefits with VCE-006.1 were associated with a reduction in the loss of TH-containing neurons caused by a 6-OHDA lesion in the substantia nigra (F(3,27) = 25.57, *p* < 0.0001; Figure 3A,B). The 6-OHDA lesion also caused a modest elevation of LAMP-1 immunostaining, a marker of autophagy, which was attenuated by the treatment with VCE-006.1 (F(3,29) = 4.77, *p* < 0.01; Figure 3C,D).

**Figure 4:**
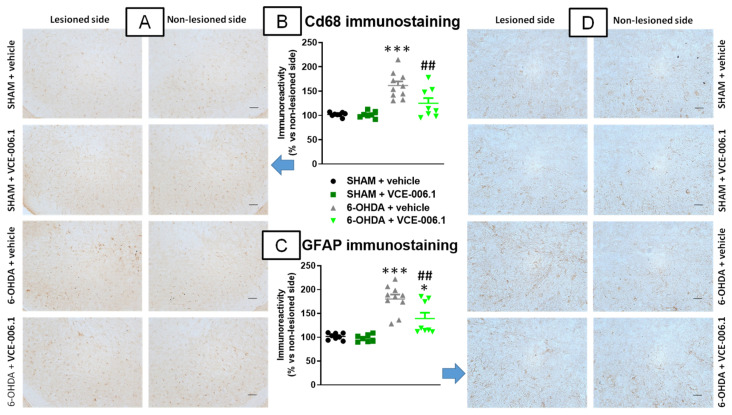
Quantification of Cd68 (**B**) and GFAP (**C**) immunoreactivities, including representative images (**A**) (Cd68; scale bar = 100 µm) and (**D**) (GFAP; scale bar = 50 µm)), measured in a selected area of the substantia nigra pars compacta of male mice subjected to unilateral 6-OHDA lesions or sham-operated and daily treated with VCE-006.1 (20 mg/kg, i.p.) for 2 weeks. Values correspond to % of the ipsilateral lesioned side vs. contralateral non-lesioned side and are expressed as means ± SEM of more than 6 animals per group. Data were assessed by one-way ANOVA followed by the Tukey test (* *p* < 0.05, *** *p* < 0.005 vs. the two sham-operated groups; ^##^ *p* < 0.01 vs. the vehicle-treated 6-OHDA lesioned mice).

Our histological analysis of the substantia nigra also proved an elevated glial reactivity detected in this structure when lesioned with 6-OHDA, visible for Cd68 immunolabelling (reflecting reactive microgliosis) and with GFAP immunostaining. Both responses were notably attenuated by the treatment with VCE-006.1 (Cd68: F(3,29) = 15.43, *p* < 0.0001; Figure 4A,B; GFAP: F(3,29) = 22.72, *p* < 0.0001; Figure 4C,D). VCE-006.1 had no effect on these markers in sham-operated mice.

**Figure 5:**
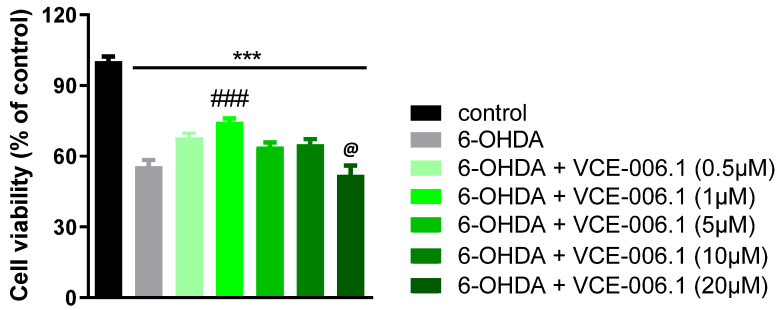
Cell viability measured with the MTT assay in cultured SH-SY5Y cells at 24 h to be treated with different concentrations of VCE-006.1 (0.5, 1, 2, 5, 10, and 20 µM) against 6-OHDA (200 µM). In all cases, a group with cells exposed to vehicle was also included to determine the 100% of cell viability. Values are means ± SEM of at least 4 independent experiments, each performed in triplicate. Data were assessed by the one-way ANOVA followed by the Tukey (*** *p* < 0.005 vs. control cells; ^###^ *p* < 0.005 vs. cells exposed to 6-OHDA + vehicle; ^@^ *p* < 0.05 vs. cells treated with the other VCE-006.1 concentrations).

In a second experiment, we investigated whether VCE-006.1 also exerts similar cytoprotective effects in vitro in SH-SY5Y cells, which express GPR55 [67], exposed to 6-OHDA. Our data revealed that 6-OHDA reduced cell viability up to close to 50% in these cells, which was attenuated by VCE-006.1 in a concentration-related manner with a maximum at 1 µM (F(6,40) = 40.80, *p* < 0.0001), lower effects at higher concentrations (5 and 10 µM), and no effect at 20 µM (Figure 5).

**Figure 6:**
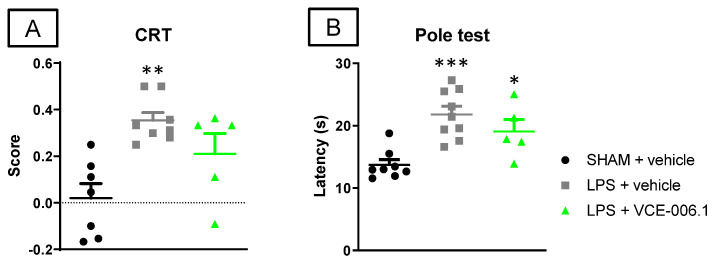
Response in the cylinder rearing test (**A**) and in the pole test (**B**) of male mice subjected to unilateral LPS lesions or sham-operated and daily treated with VCE-006.1 (20 mg/kg, i.p.) for 2 weeks. Values are means ± SEM of more than 6 animals per group. Data were assessed by one-way ANOVA followed by the Tukey test (* *p* < 0.05, ** *p* < 0.01, *** *p* < 0.005 vs. the two sham-operated groups).

Next, we also investigated VCE-006.1 in an inflammatory model of PD, LPS-lesioned mice, having relatively similar beneficial effects. Again, LPS-lesioned mice exhibited motor defects in the cylinder rearing test (hemiparesis) and in the pole test (elevated latency to descend the pole), which were attenuated by the treatment with VCE-006.1 (CRT: F(2,17) = 9.34, *p* < 0.005; Figure 6A; pole test: F(2,19) = 11.75, *p* < 0.0005; Figure 6B).

**Figure 7:**
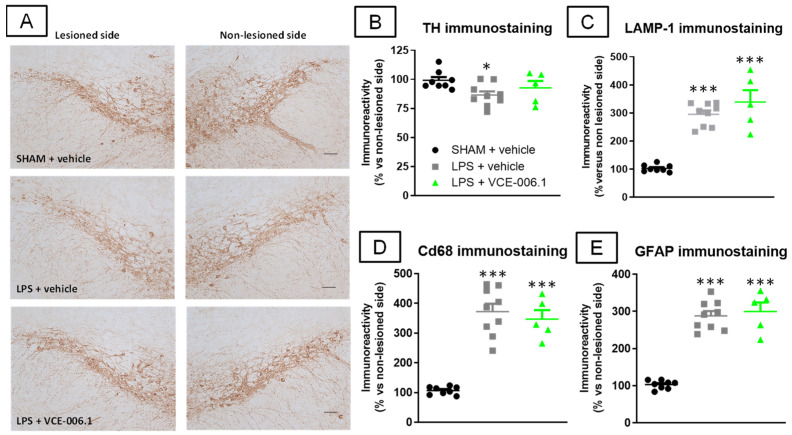
Quantification of TH (**B**), LAMP-1 (**C**), Cd68 (**D**), and GFAP (**E**) immunoreactivities, including representative images for TH immunostaining ((**A**); scale bar = 100 µm), measured in a selected area of the substantia nigra pars compacta of male mice subjected to unilateral LPS lesions or sham-operated and daily treated with VCE-006.1 (20 mg/kg, i.p.) for 2 weeks. Values correspond to % of the ipsilateral lesioned side vs. contralateral non-lesioned side and were expressed as means ± SEM of more than 5 animals per group. Data were assessed by one-way ANOVA followed by the Tukey test (* *p* < 0.05, *** *p* < 0.005 vs. the two sham-operated groups).

These benefits of VCE-006.1 on the neurological state of LPS-lesioned mice were again accompanied by higher survival or TH-positive neurons in the substantia nigra (F(2,19) = 3.45, *p* < 0.05; Figure 7A,B), an effect that was modest and reflected in the loss of statistically significant differences vs. sham-operated animals. However, this effect, surprisingly, was not accompanied by a reduction in the LPS-induced elevation of the autophagy marker LAMP-1 (F(2,19) = 42.56, *p* < 0.0001; Figure 7C). The same happened with the reactive microgliosis (elevated Cd68 immunoreactivity; F(2,19) = 45.80, *p* < 0.0001; Figure 7D) and astroglial reactivity (elevated GFAP immunolabelling; F(2,19) = 69.94, *p* < 0.0001; Figure 7E), which remained elevated in LPS-lesioned mice irrespective of VCE-006.1 treatment.

**Figure 8:**
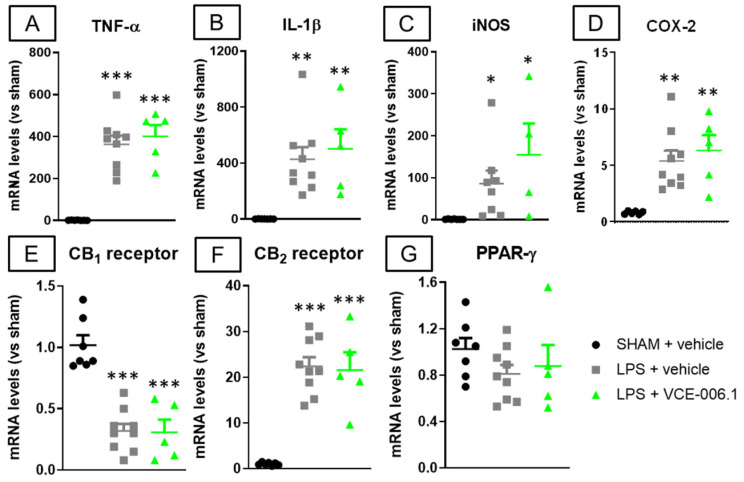
mRNA levels for TNF-α (**A**), IL-1β (**B**), iNOS (**C**), COX-2 (**D**), CB_1_ receptor (**E**), CB_2_ receptor (**F**), and PPAR-γ (**G**) measured by qPCR in the striatum of male mice subjected to unilateral LPS lesions or sham-operated and daily treated with VCE-006.1 (20 mg/kg, i.p.) for 2 weeks. GAPDH was used as an endogenous reference gene for data normalization. Values correspond to fold of change vs. sham-operated controls and are expressed as means ± SEM of more than 5 animals per group. Data were assessed by one-way ANOVA followed by the Tukey test (* *p* < 0.05, ** *p* < 0.01, *** *p* < 0.005 vs. the two sham-operated groups).

Such absence of VCE-006.1 effects against glial reactivity was also evident against some associated inflammatory events elicited by LPS lesion, for example the elevated gene expression detected in the striatum in proinflammatory cytokines TNF-α (F(2,18) = 33.34, *p* < 0.0001; Figure 8A) and IL-1β (F(2,18) = 9.41, *p* < 0.005; Figure 8B), as well as in proinflammatory enzymes iNOS (F(2,16) = 4.24, *p* < 0.05; Figure 8C) and COX-2 (F(2,17) = 9.13, *p* < 0.005; Figure 8D), which remained unaltered after VCE-006.1 treatment. This was also evident for the LPS-induced reduction in the CB_1_ receptor (F(2,18) = 28.63, *p* < 0.0001; Figure 8E), elevation of the CB_2_ receptor (F(2,18) = 31.31, *p* < 0.0001; Figure 8F), and no effect in PPAR-γ (F(2,18) = 1.14, ns; Figure 8G)

**Figure 9:**
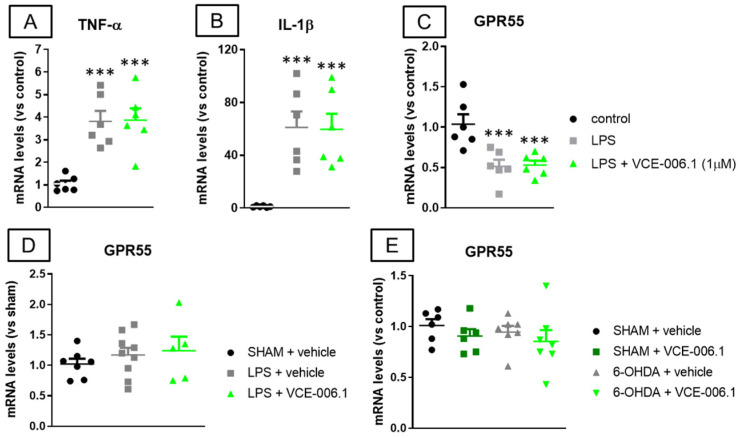
mRNA levels for TNF-α (**A**), IL-1β (**B**), and GPR55 (**C**) measured by qPCR in BV2 cells exposed to LPS and/or VCE-006.1 (1 µM) for 20 h, and mRNA levels for GPR55 measured by qPCR in the striatum of male mice subjected to unilateral 6-OHDA (**D**) or LPS (**E**) lesions or sham-operated and daily treated with VCE-006.1 (20 mg/kg, i.p.) for 2 weeks. In all cases, GAPDH was used as an endogenous reference gene for data normalization, and values correspond to fold change vs. controls and are expressed as means ± SEM of more than 5 animals per group. Data were assessed by one-way ANOVA followed by the Tukey test (*** *p* < 0.005 vs. the control group).

Lastly, the absence of VCE-006.1 effects against glial reactivity and associated inflammatory events detected in LPS-lesioned mice was also confirmed in BV2 cells (which also express GPR55 [68]) treated with LPS and VCE-006.1, as the elevated levels of gene expression detected for TNF-α (F(2,15) = 15.14, *p* < 0.0005; Figure 9A) and IL-1β (F(2,15) = 12.21, *p* < 0.001; Figure 9B) after LPS again remained unaltered by the treatment with VCE-006.1. This may be related to the strong reduction in GPR55 mRNA levels found in BV2 cells treated with LPS in the absence or presence of VCE-006.1 in comparison with control cells (F(2,15) = 10.53, *p* < 0.005; Figure 9C). However, the analysis of gene expression for GPR55 in the striatum of LPS-lesioned mice proved no changes in this receptor (F(2,18) = 0.57, ns; Figure 9D), and the same happened in 6-OHDA-lesioned mice (F(3,22) = 0.65, ns; Figure 9E).

### 2.3. Studies in Experimental ALS

**Figure 10:**
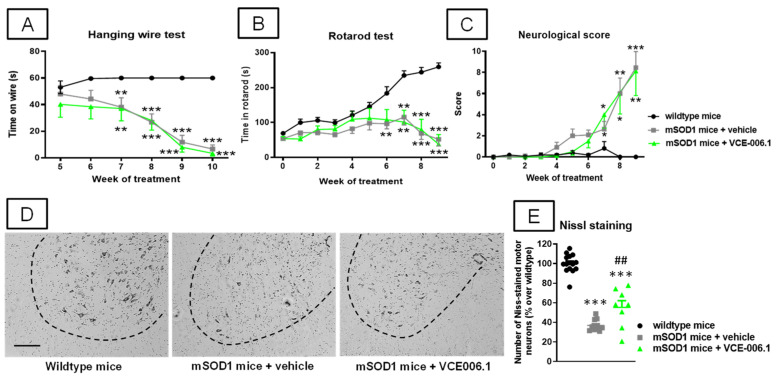
Hanging wire response (**A**), rotarod performance (**B**), and neurological score (**C**), analyzed mSOD1 transgenic and wild-type male mice at specific weeks during a chronic treatment from 63 day-old to 125 day-old with VCE-006.1 (20 mg/kg, daily and i.p.) or vehicle, and quantification of the number of Nissl-stained motor neurons (**E**), including representative images ((**D**); scale bar = 100 µm), in the lumbar ventral horn (marked with a dotted line) of the spinal cord in all experimental groups after the chronic treatment. Values are means ± SEM of more than 6 animals per group. Behavioral data were assessed by two-way ANOVA (with repeated measures), whereas Nissl staining data were assessed by one-way ANOVA, in both cases followed by the Tukey test (* *p* < 0.05, ** *p* < 0.01, *** *p* < 0.005 vs. wild-type mice; ^##^ *p* < 0.01 vs. mSOD1 mice treated with vehicle).

**Figure 11:**
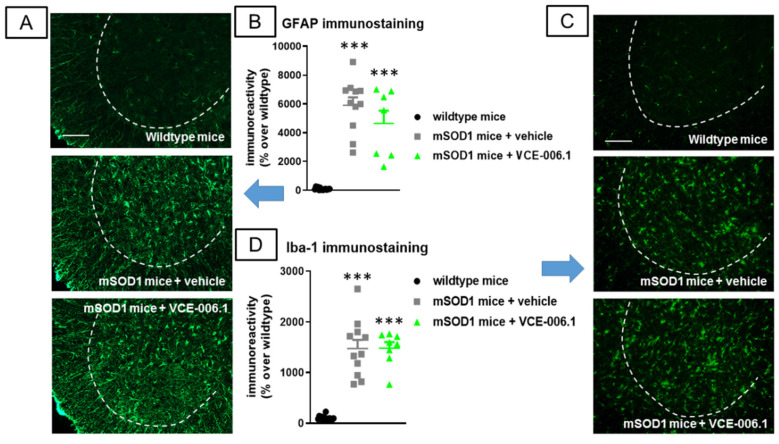
Quantification of GFAP (**B**) and Iba-1 (**D**) immunoreactivities, including representative images ((**A**) and (**C**), respectively; scale bar = 100 µm), in the lumbar ventral horn (marked with a dotted line) of the spinal cord in wild-type and mSOD1 transgenic mice after a chronic treatment from 63 day-old to 125 day-old with VCE-006.1 (20 mg/kg, daily and i.p.) or vehicle. Values are means ± SEM of more than 6 animals per group. Data were assessed by one-way ANOVA followed by the Tukey test (*** *p* < 0.005 vs. wild-type mice).

Our third objective was to investigate VCE-006.1 when administered chronically in two genetic murine models of ALS. We first used the classic mSOD-1 model which showed several motor abnormalities such as: (i) a progressive reduction in the time on wire (2-way interaction: F(10,155) = 13.25, *p* < 0.0001; Figure 10A) visible in the hanging wire test; (ii) a progressively marked deterioration in the rotarod performance (2-way interaction: F(18,270) = 15.43, *p* < 0.0001; Figure 10B) detected in the rotarod test; and (iii) a rapid elevation in a specific neurological score for ALS signs recapitulated in mice (2-way interaction: F(18,288) = 10.23, *p* < 0.0001; Figure 10C). VCE-006.1 was not active against any of these neurological decline signs, then indicating no effects at the functional level. However, the strong loss of Nissl-stained motor neurons visible in the ventral horn of the spinal cord (lumbar levels) in mSOD-1 mice was partially attenuated by the chronic treatment with VCE-006.1 (F(2,31) = 98.79, *p* < 0.0001; Figure 10D,E), although this does not have any influence on possible neurological recoveries as seen in the above behavioral data. This may be in part related to the persistence of higher levels of glial reactivity in the ventral horn of the spinal cord (lumbar levels) in mSOD-1 mice after the treatment with VCE-006.1 (GFAP immunolabelling: F(2,30) = 53.34, *p* < 0.0001; Figure 11A,B); Iba-1 immunolabelling: F(2,31) = 62.56, *p* < 0.0001; Figure 11C,D), which were similar to mSOD-1 mice treated with vehicle.

**Figure 12:**
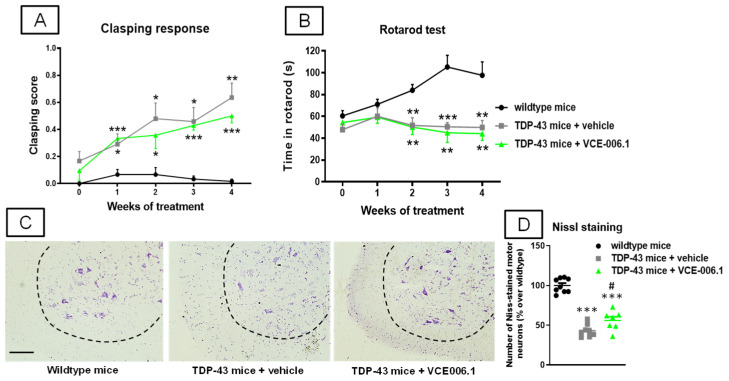
Clasping response (**A**) and rotarod performance (**B**) analyzed TDP-43 transgenic and wild-type male mice at specific weeks during a chronic treatment of 30 days with VCE-006.1 (20 mg/kg, daily and i.p.) or vehicle, and quantification of the number of Nissl-stained motor neurons (**D**), including representative images ((**C**); scale bar = 100 µm), in the lumbar ventral horn (marked with a dotted line) of the spinal cord in all experimental groups after the chronic treatment. Values are means ± SEM of more than 6 animals per group. Behavioral data were assessed by two-way ANOVA (with repeated measures), whereas Nissl staining data were assessed by one-way ANOVA, in both cases followed by the Tukey test (* *p* < 0.05, ** *p* < 0.01, *** *p* < 0.005 vs. wild-type mice; ^#^ *p* < 0.05 vs. TDP-43 mice treated with vehicle).

Next, we investigated the same issue in an alternative and more recent ALS model based on the RNA-binding protein TDP-43. Again, TDP-43 transgenic mice showed several motor abnormalities such as: (i) a progressively higher clasping response (2-way interaction: F(8,88) = 4.50, *p* < 0.0001; Figure 12A); and (ii) a progressively marked deterioration in the rotarod performance (2-way interaction: F(8,88) = 4.46, *p* < 0.0001; Figure 12B) detected in the rotarod test. Again, VCE-006.1 was not active against any of these motor signs, then indicating no effects at the functional level, despite the strong loss of Nissl-stained motor neurons visible in the ventral horn of the spinal cord (lumbar levels) in TDP-43 transgenic mice was partially attenuated by the chronic treatment with VCE-006.1 (F(2,21) = 82.28, *p* < 0.0001; Figure 12C,D).

**Figure 13:**
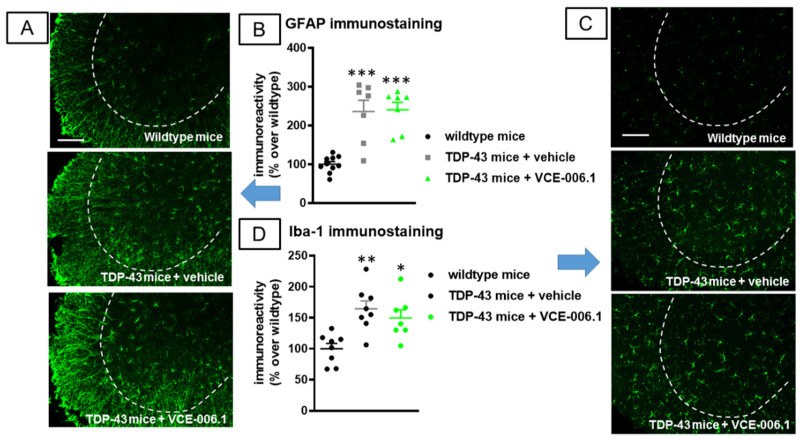
Quantification of GFAP (**B**) and Iba-1 (**D**) immunoreactivity, including representative images ((**A**,**C**), respectively; scale bar = 100 µm), in the lumbar ventral horn (marked with a dotted line) of the spinal cord in wild-type and TDP-43 transgenic mice after chronic treatment of 30 days with VCE-006.1 (20 mg/kg, daily and i.p.) or vehicle. Values are means ± SEM of more than 6 animals per group. Data were assessed by one-way ANOVA followed by the Tukey test (* *p* < 0.05, ** *p* < 0.01, *** *p* < 0.005 vs. wildtype mice).

Again, we may attribute this effect in part to the persistence of higher levels of glial reactivity in the ventral horn of the spinal cord (lumbar levels) in TDP-43 transgenic mice after the treatment with VCE-006.1 (GFAP immunolabelling: F(2,21) = 21.08, *p* < 0.0001; Figure 13A,B); Iba-1 immunolabelling: F(2,20) = 8.82, *p* < 0.005; Figure 13C,D), which were similar to TDP-43 transgenic mice.

**Figure 14:**
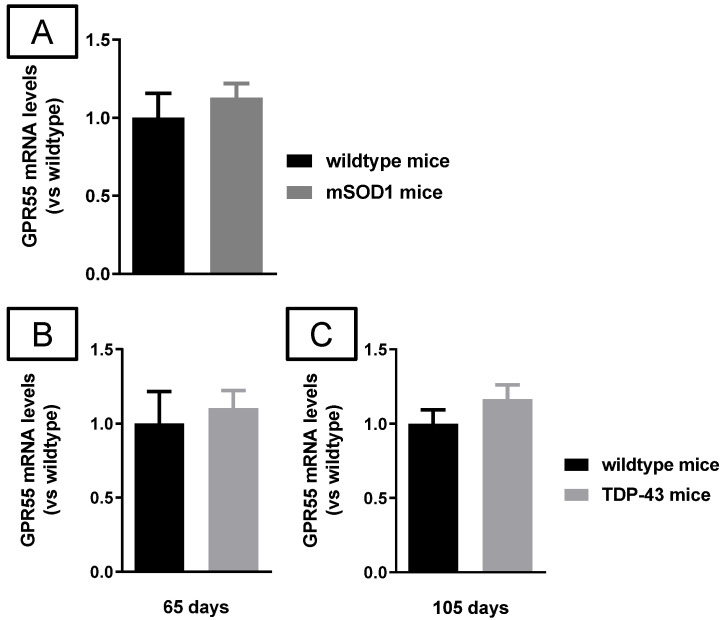
mRNA levels for GPR55 measured by qPCR in the spinal cord of male mSOD1 (at 123 days of age; (**A**)) or TDP-43 (at 65 (**B**) and 105 (**C**) days of age) transgenic mice, and their corresponding wild-type mice. GAPDH was used as an endogenous reference gene for data normalization. Values correspond to fold change vs. controls and are expressed as means ± SEM of more than 5 animals per group. Data were assessed by the unpaired Student’s *t*-test.

Lastly, as in the experimental models of PD, we also analyzed GPR55 gene expression in these in vivo ALS models. Our data indicated that GPR55-mRNA levels did not experience any changes in the case of mSOD1 mice compared to wild-type animals when analyzed at a late symptomatic phase (123 days; Figure 14A), and the same happened with TDP-43 transgenic mice at two specific ages: 65 (early symptomatic stage; Figure 14B) and 105 days (advanced symptomatic phase; Figure 14C).

## 3. Discussion

The orphan receptor GPR55 has emerged in the last years as a potential new component of the endocannabinoid signaling system [54], despite its differences with the classic CB_1_ and CB_2_ receptors [55,56,57], as well as a promising neuroprotective target for the development of novel therapies for neurodegenerative conditions [48,49,50,51,52]. One of the key areas, involving GPR55 activity in the CNS, is the control of movement and motor coordination, which is supported by the fact that motor-related areas (e.g., basal ganglia, cerebellum) are within the CNS structures with higher GPR55 expression [63]. In addition, GPR55-deficient mice develop, among others, important impairments in motor control and coordination [53]. This possibly explains that neurodegenerative disorders such as Alzheimer’s disease and related dementias have been explored for determining the neuroprotective potential of GPR55-targeting compounds only recently [69,70], whereas movement-related disorders, in particular PD, are within those neurodegenerative pathologies investigated earlier and more extensively in relation with the GPR55 ligands [51,52,71,72]. Our present study has been designed to pursue the objective of developing a GPR55-based neuroprotective therapy for PD and also by other motor-related pathologies, for example, ALS. To do that, we used a chromenopyrazole derivative, VCE-006.1, which a priori showed selective properties as a partial agonist at the GPR55 receptors [65]. Our first objective was to extend the characterization of this compound to its activity at the GPR55 receptor, using specific cell assays that revealed a biased activity of VCE-006.1 on this receptor as a partial orthosteric agonist or PAM, depending on the specific cell assay used.

Once we confirmed this activity of VCE-006.1 at the GPR55 receptor, we wanted to explore whether this enables the compound to afford neuroprotection in cells and murine models of the two neurodegenerative diseases indicated before, i.e., PD and ALS. Our experiments in PD demonstrated that VCE-006.1 was highly active in the preservation of TH-containing nigral neurons damaged in this disease, and that this has an important reflect in the improvement of motor defects associated with this damage. In our study, this neuroprotective effect was evident in two in vivo models of PD generated by 6-OHDA or, to a lower extent, LPS lesions in mice, and was also confirmed in an in vitro cell-based model (SH-SY5Y cells exposed to 6-OHDA). Similar benefits have been observed with other GPR55-acting compounds using additional experimental models, such as MPTP-lesioned mice and a murine model of haloperidol-induced catalepsy [51], and the same happens with more recent studies conducted by Martínez-Pinilla and coworkers [52,71]. However, whereas the neuroprotection seen in 6-OHDA-lesioned mice with VCE-006.1 in our study was accompanied by an attenuation of the reactive gliosis elicited by the neurotoxin, this did not occur in the LPS-lesioned mice, in which the inflammatory response caused by LPS has been proposed to be the primary cause of further neuropathological events (e.g., loss of TH-positive neurons, motor defects). These paradoxical effects remain to be investigated, but, in support of this in vivo effect, the lack of VCE-006.1 effect against glial reactivity and associated inflammatory events (elevated generation of proinflammatory cytokines) was also evident in BV2 cells treated with LPS and VCE-006.1. This could be related to an LPS-induced down-regulation of GPR55 receptors in the BV2 cells, although such down-regulation was not found in LPS-lesioned mice, and the same was seen in 6-OHDA-lesioned mice. In addition, in preliminary studies carried out with post mortem tissues from PD patients and control subjects, we detected apparently similar levels of GPR55 and an equivalent cell distribution, although this will require further confirmation (García, Burgaz and Fernández-Ruiz, unpublished results). To make the issue more complicated and justify the need for additional studies, a previous experiment also conducted in BV2 cells, and in part in rat microglial cell primary cultures, showed activity of LPI against LPS-induced nitric oxide production and iNOS expression [50]. By contrast, a similar study was carried out with anandamide, which also binds GPR55; instead, LPI resulted in inactivity [73].

As indicated before, we also investigated VCE-006.1 in another motor-related neurodegenerative disorder, ALS, using two genetic models of this pathology, the classic mSOD-1 model and the more recent TDP-43 transgenic mice. In both cases, our results confirmed that VCE-006.1 was poorly active, exerting only partial preservation of spinal motor neurons, which was not sufficient to reverse the intense neurological decline and muscle strength deterioration seen in these animals during the progression of the pathological phenotype. This may be related to the lack of effect of VCE-006.1 on the elevated microglial and astroglial reactivities seen in both models, a fact that, in this case, was not associated with a reduction in the levels of GPR55 receptors, which resulted in being similar to those found in the corresponding wild-type mice for both TDP-43 transgenic and mSOD-1 mice. Combining neuroprotection (preservation of motor neurons) and anti-inflammatory (attenuation of glial reactivity) effects appear to be an important determinant for disease-modifying effects of cannabinoids in experimental ALS. For example, cannabinoids targeting the CB_2_ (e.g., HU-308) or the PPAR-γ receptors (e.g., VCE-003.2) afforded important levels of neuroprotection, being able to preserve motor neurons and to attenuate glial reactivity, which results in an improvement against the neurological (motor) deterioration [30,32,45]. However, such neurological improvement was not observed in studies that used cannabinoids that were not active at the same time against both the loss of motor neurons and the elevated glial reactivity [74]. Therefore, we assume that the potential of VCE-006.1 for ALS would require its combination with other cannabinoids also active at other endocannabinoid-related targets (e.g., CB_2_ receptors, PPAR-γ receptors). We also have evidence that VCE-006.1 does not activate PPAR-γ receptors (Muñoz et al., unpublished results).

## 4. Materials and Methods

### 4.1. Synthesis and Characterization as PAM of VCE-006.1 in Cell-Based Assays

VCE-006.1 (2-[2-(4-cyclohexylcarbonylpiperazinyl)ethyl]-2,4-dihydro-7-methoxy-4,4-dimethylchromeno[4,3-c]pyrazole) was designed, synthesized, and characterized for the first time as a partial agonist at the GPR55 receptor by Morales and coworkers (compound 23 in [65]). In this new study, we have further characterized its biological activity profile both in HEK-293 cells overexpressing GPR55 and in cell lines expressing the native receptor.

#### 4.1.1. Determination of ERK 1/2 Activation

DU145 cells expressing endogenous GPR55 were stimulated with either VCE-006.1 (5 µM), LPI (2 µM), or a combination of both for 30 min. Then, cells were washed with phosphate-buffered saline (PBS) and proteins extracted in lysis buffer (50 mM Tris–HCl pH 7.5, 150 mM NaCl, 10% glycerol, and 1% NP-40) supplemented with 10 mM NaF, 1 mM Na_3_VO_4_, 10 μg/mL leupeptin, 1 μg/mL pepstatin and aprotinin, and 1 μL/mL saturated PMSF. Thirty μg of proteins were boiled at 95 °C in Laemmli buffer and electrophoresed in 10% SDS/PAGE gels. Total ERK was used as a loading control. Separated proteins were transferred to PVDF membranes, and after blocking with non-fat milk in TBST buffer, primary antibodies were added. The washed membranes were incubated with appropriate secondary antibodies coupled to horseradish peroxidase that were detected by an enhanced chemiluminescence system (USB). Antibodies against total and phospho-ERK1/2 were purchased from Sigma-Aldrich (Madrid, Spain).

#### 4.1.2. Ca^2+^ Mobilization Assay

U937 cells expressing endogenous GPR55 receptor were incubated for 1 h at 37 °C in Tyrode’s salt solution (137 mM NaCl, 2.7 mM KCl, 1.8 mM CaCl_2_, 1.0 mM MgCl_2_, 0.4 mM NaH_2_PO_4_, 12.0 mM NaHCO_3_, and 5.6 mM D-glucose) containing 5 µM Indo1-AM (Invitrogen, Waltham, MA, USA) for 30 min at 37 °C in the dark. Cells were then harvested, washed three times with buffer to remove extracellular Indo1 dye, readjusted to 10^6^ cells/mL in the appropriate buffer, and analyzed in a spectrofluorometer operated in the ratio mode (model F-2500; Hitachi Ltd., Tokyo, Japan) under continuous stirring and at a constant temperature of 37 °C using a water-jacketed device. After a 5-min accommodation to equilibrate temperatures, samples were excited at 338 nm, and emission was collected at 405 and 485 nm, corresponding to the fluorescence emitted by Ca^2+^ bound and -free Indo1, respectively. The cells were stimulated with either LPI or VCE-006.1, and maximal ratio values for calculations were determined by the addition at the end of the measurements of 10 µM ionomycin. [Ca^2+^]i changes are presented as changes in the ratio of bound to free calcium (340 nm/380 nm).

#### 4.1.3. cAMP Signaling Induced by GPR55 Activation

The determination of GPR55 activity was carried out using the HEK293T-GPR55 cells stably transfected with the human GPR55 cDNA. Briefly, HEK293T-GPR55 cells were transiently transfected with 0.2 µg of the reporter plasmid CRE-Luc that contains six consensus cAMP-responsive elements (CRE) linked to the firefly luciferase reporter gene using Roti©-Fect (Carl Roth, Karlsruhe, Germany). Transfected cells were treated with either VCE-006.1, LPI, or a combination of both. After 6 h of stimulation, cells were washed twice with PBS 1× and lysed in 100 µL lysis buffer containing 25 mM Tris-phosphate (pH 7.8), 8 mM MgCl_2_, 1 mM DTT, 1% Triton X-100, and 7% glycerol for 15 min at room temperature in a horizontal shaker. Luciferase activity was measured using a TriStar2 Berthold/LB942 multimode reader (Berthold Technologies, Bad Wildbad, Germany) following the instructions of the luciferase assay kit (Promega, Madison, WI, USA). The RLUs (relative light units) were calculated, and the results were expressed as fold induction over unstimulated cells. The experiment was performed 5–6 times.

### 4.2. Animals and Cell Experiments

#### 4.2.1. PD Experiments

Male C57BL/6 mice were housed in a room with a controlled photoperiod (08:00–20:00 light) and temperature (22 ± 1 °C). They had free access to standard food and water and were used at adult age (3–4 month-old; 25–30 g weight). All experiments were conducted according to national and European guidelines (directive 2010/63/EU), as well as conformed to ARRIVE guidelines and approved by the “Comité de Experimentación Animal” of our university (PROEX: 056/19).

In a first experiment, male C57BL/6 mice were subjected to stereotaxic unilateral application of 6-hydroxydopamine (6-OHDA) or saline [24,75]. To do that, mice were anesthetized (ketamine 40 mg/kg + xylazine 4 mg/kg, i.p.) 30 min after pretreatment with desipramine (25 mg/kg, i.p.), and then 6-OHDA free base (2 μL at a concentration of 2 μg/μL saline in 0.2% ascorbate to avoid oxidation) or saline (for control mice) were injected stereotaxically into the right striatum at a rate of 0.5 μL/min, using the following coordinates: + 0.4 mm AP, −1.8 mm ML and −3.5 mm DV, as described in [75]. Once injected, the needle was left in place for 5 min before being slowly withdrawn, thus avoiding reflux and a rapid increase in intracranial pressure. Control animals were sham-operated and injected with 2 μL of saline using the same coordinates. The lesions were generated using unilateral injection, the advantage of which is that contralateral structures serve as controls for the different analyses. After the application of 6-OHDA or saline, animals were subjected to a daily treatment with VCE-006.1 (20 mg/kg, i.p.) or vehicle (cremophor-saline, 1:18) for two weeks, at the end of which (24 h after the last injection), they were analyzed in the pole test and the cylinder rearing test just before being killed by rapid and careful decapitation and their brains rapidly removed. Brains were divided coronally into two parts, following the procedure described by Palkovits and Brownstein [76]. The anterior halves were used to dissect the striatum (both ipsilateral and contralateral sides separately), and tissues were rapidly frozen by immersion in cold 2-methylbutane and stored at −80 °C for qPCR analysis. The posterior halves containing the midbrains were fixed for one day at 4 °C in fresh 4% paraformaldehyde prepared in 0.1 M PBS, pH 7.4. Samples were cryoprotected by immersion in a 30% sucrose solution for a further day, and finally stored at −80 °C for immunohistochemical analysis in the substantia nigra.

In a second experiment, mice were anesthetized (ketamine 40 mg/kg + xylazine 4 mg/kg, i.p.) and subjected to unilateral injections of *S. Minnesota* LPS (Sigma-Aldrich, Madrid, Spain) into two points of the right striatum following the procedure developed by Hunter et al. [77]. We used the following stereotaxic coordinates from bregma: + 1.1 mm AP, −1.8 mm ML, and −3.5 mm DV, as well as −0.3 mm AP, −2.5 mm ML, and −3.2 mm DV (see details in [77]). At each intrastriatal coordinate, 5 μg of LPS in a volume of 1 μL of saline was injected slowly (0.5 μL/30 s), and the needle was again left in place for 5 min before being slowly withdrawn. This avoids generating reflux and a rapid increase in intracranial pressure. Control animals were sham-operated and injected with 1 μL of saline using the same coordinates. Again, the lesions were generated using unilateral administration, the advantage of which is that contralateral structures serve as controls for the different analyses. After the application of LPS or saline, animals were subjected to a daily treatment with VCE-006.1 (20 mg/kg, i.p.) or vehicle (cremophor-saline, 1:18) for two weeks, at the end of which (24 h after the last injection), they were analyzed in the pole test and the cylinder rearing test just before being killed by rapid and careful decapitation and their brains rapidly removed and processed as described before 6-OHDA-lesioned mice.

In a third experiment, cultures of SH-SY5Y neuronal cell line (kindly provided by Dr. Ana Martínez, CIB-CSIC, Madrid, Spain) were used to induce cell death with 6-OHDA and to investigate in vitro the possible cytoprotective effects of VCE-006.1, following a procedure described previously [78]. To this end, SH-SY5Y cells were maintained in Dulbecco’s Modified Eagle’s Medium (DMEM; Lonza, Verviers, Belgium) supplemented with 10% fetal bovine serum (FBS), 2 mM Ultraglutamine, and 1% antibiotics (Lonza, Verviers, Belgium) under a humidified 5% CO_2_ atmosphere at 37 °C. For cytotoxicity experiments, cells were seeded at 60,000 cells/well in 96-well plates and maintained under a humidified atmosphere (5% CO_2_) at 37 °C overnight. For experiments, 24 h after seeding, cells were treated with the vehicle (DMEM + 0.1% DMSO) or with five different concentrations of VCE-006.1 (0.5, 1, 2, 5, 10, and 20 μM; selected according to [65]), 60 min before being exposed to 200 µM 6-OHDA (or saline) following our previously published studies with different concentrations of 6-OHDA in these cells [43,44]. Cells were incubated 24 h before the neuronal death was analyzed with the MTT assay (Panreac AppliChem., Barcelona, Spain). Data of cell viability were normalized in relation to the corresponding control group (cells exposed to vehicles for 6-OHDA and VCE-006.1).

In a fourth experiment, cultured BV-2 cells were maintained in DMEM (Lonza, Verviers, Belgium) supplemented with 10% FBS (Sigma-Aldrich, Madrid, Spain), 2 mM Ultraglutamine, and antibiotics (Lonza, Verviers, Belgium) in a humidified atmosphere of 5% CO_2_ at 37 °C. Cells were plated at a density of 45 × 10^4^ cells per well in 12-well culture plates and incubated in DMEM with a reduction of FBS to 1%. Three hours later, cells were treated with 0.5 μg/mL LPS (from *Escherichia coli* 055:B5, Sigma-Aldrich, Madrid, Spain), alone or in combination with VCE-006.1, used at a concentration of 1 μM, and added 1 h before LPS. Twenty hours after the addition of LPS, media were removed, and cell pellets were collected for analyzing mRNA levels of GPR55, tumor necrosis factor-α (TNF-α), and interleukin-1β (IL-1β) using qPCR analysis.

#### 4.2.2. ALS Experiments

Experiments were conducted with two mouse colonies: (i) B6SJL-Tg(SOD1*G93A)1Gur/J transgenic (mSOD1 mice) and non-transgenic littermate sibling mice bred in our animal facilities from initial breeders provided by Dr. Rosario Osta (LagenBio-Ingen, University of Zaragoza, Spain), and (ii) Prp-hTDP-43(A315T) transgenic and non-transgenic littermate sibling mice bred in our animal facilities from initial breeders purchased from Jackson Laboratories (Bar Harbor, ME, USA). In both cases, animals were subjected to genotyping for identifying the presence or absence of the transgene containing the SOD-1 or the TDP-43 mutation (see details in [30,45], respectively). As in PD experiments, all animals were housed in a room with controlled photoperiod (08:00–20:00 light) and temperature (22 ± 1 °C) with free access to standard food or, in the case of TDP-43 transgenic mice, to a high-fat jelly diet (DietGel Boost, ClearH20, Portland, ME, USA) [79], and water. All experiments were conducted according to local and European rules (directive 2010/63/EU), as well as conformed to ARRIVE guidelines. They were approved by the ethical committees of our university and the regulatory institution (PROEX: 056/19).

In a first experiment, wild-type and mSOD-1 transgenic mice were identified by numbered ear marks, and prior to the start of the different experiments, they were randomly allocated to the different treatment groups. We treated B6SJL-Tg(SOD-1*G93A)1Gur/J transgenic male mice with VCE-006.1, synthesized as previously described [65], and administered i.p. to mice at the dose of 20 mg/kg. Additional transgenic mice, as well as wild-type animals, were treated with vehicle (cremophor-saline, 1:18). The treatment was initiated when animals were 63 days old and prolonged daily up to the age of 18 weeks (125 days of age). During this period, animals were weighed every day and subjected to several neurological analyses and behavioral tests at specific time points. Twenty-four hours after the last injection, animals were euthanized by rapid decapitation, and their spinal cords were dissected and removed. The spinal cords (lumbar level) to be used for histology were fixed for one day at 4 °C in 4% formaldehyde solution in PBS. Samples were then cryoprotected by immersion in a 30% sucrose solution for a further day, and finally stored at −80 °C for Nissl staining and immunohistochemical analysis. The spinal samples (also lumbar area) to be used for qPCR analyses were collected and rapidly frozen by immersion in cold 2-methylbutane and stored at −80 °C for qPCR analysis.

In a second experiment, we treated non-transgenic and Prp-hTDP-43(A315T) transgenic male mice with VCE-006.1, again synthesized as previously described [65] and administered i.p. to mice at the dose of 20 mg/kg. Additional transgenic mice, as well as wild-type animals, were treated with vehicle (cremophor-saline, 1:18). The treatment was initiated when animals were 65 days old and prolonged daily up to the age of 95 days, the same treatment window used in our previous study [30], which extends from early symptomatic phases (around the 9th week of age) up to an advanced stage (around the 13th week of age). Animal weight was logged daily. Weight loss of 20% was established as the human end-point. Rotarod performance and clasping reflex to detect dystonia were recorded weekly during the 4 weeks of the treatment period (including a recording just before the first injection). All animals were euthanized by rapid decapitation at the age of 95 days, at least 24 h after the last administration. Their spinal cords were rapidly removed and processed as described for mSOD-1 mice.

### 4.3. Behavioral Recording

#### 4.3.1. Pole Test

Mice were placed head-upward on the top of a vertical rough-surfaced pole (diameter 8 mm; height 55 cm), and the time until animals descended to the floor was recorded with a maximum duration of 120 s. When the mouse was not able to turn downward and instead dropped from the pole, the time was taken as 120 s (default value) (see details in [44]).

#### 4.3.2. Cylinder Rearing Test

Given that the lesion was unilateral in the experiment with 6-OHDA or LPS, this test attempted to quantify the degree of forepaw (ipsilateral, contralateral, or both) preference for wall contacts after placing the mouse in a methacrylate transparent cylinder (diameter: 15.5 cm; height: 12.7 cm [80]). Each score was made out of a 3 min trial with a minimum of 4 wall contacts.

#### 4.3.3. Neurological Score

Mice were evaluated for neurological decline using a numerical scale published previously [45]. The scale ranged from 0 to 15 distributed in three sub-scales (0–5) concentrated on ambulation, strength analysis, and hind-foot reflex test. A final score of 0 corresponds to animals that are not symptomatic, whereas a score of 15 reflects a state of total functional loss in hindlimbs and postural control. The assessment of ambulation was carried out by placing the animal inside a corridor (10 × 10 × 80 cm) while evaluating postural control and the way in which hindlimbs were leaned during motion. The strength test evaluated the animal’s ability to drag and offer resistance when the tail was pulled softly to the opposite direction in which the animal moves. Lastly, the hind-foot reflex test evaluated the stiffness of the limbs and their coordination when the mouse was suspended by the tail 10 cm over the surface. The final score was calculated from the sum of values reached in each sub-scale.

#### 4.3.4. Rotarod Test

Mice were evaluated for possible motor weakness using the rotarod test, using an LE8200 device (Panlab, Barcelona, Spain). Mice were exposed to a period of acclimation and training (first session: 0 r.p.m. for 30 s; second and third sessions: 4 r.p.m. for 60 s, with periods of 10 min between sessions), followed 30 min later by the assay. Mice were placed into the apparatus, and the rotational speed was increased from 4 to 40 r.p.m. over a period of 300 s to measure the time to fall off. Mice were tested for 3 consecutive trials with a rest period of approximately 15 min between trials, and the mean of the 3 trials was calculated.

#### 4.3.5. Clasping Response

Dystonia was evaluated by picking up the mouse by the base of the tail for 30 s so that the mouse was facing downwards away from any object. The position of the hindlimbs was observed and scored following the scale reported by Guyenet et al. [81]. Animals were scored as follows: 0 if the hindlimbs were consistently extended away from the abdomen; 1 if one hindlimb was retracted toward the abdomen; 2 if both hindlimbs were partially retracted toward the abdomen; 3 if both hindlimbs were entirely retracted and touching the abdomen. Mice were tested for three consecutive trials, and the mean clasping score of the three trials was calculated.

#### 4.3.6. Hanging Wire Test

The latency of mice to fall from a wire cage top, which was slowly inverted and suspended at approximately 30 cm to the floor, was also used as an index of motor weakness. The test was repeated three times to obtain the mean value of the three trials.

### 4.4. Histological Procedures

#### 4.4.1. Tissue Slicing

In the PD experiment, brains were sliced in coronal sections (containing the substantia nigra) in a cryostat (30 µm thick) and collected on antifreeze solution (glycerol/ethylene glycol/PBS; 2:3:5) and stored at −20 °C until used for immunostaining. In the ALS experiment, fixed spinal cords were sliced with a cryostat at the lumbar level (L4-L6) to obtain coronal sections (20 μm thick) that were collected on gelatin-coated slides. Sections were used for procedures of Nissl-staining and immunostaining.

#### 4.4.2. Immunohistochemistry Analysis in the PD Experiment

Brain sections containing the substantia nigra were mounted on gelatin-coated slides and, once adhered, washed in 0.1 M potassium PBS (KPBS) at pH 7.4. Endogenous peroxidase was blocked by 30 min incubation at room temperature in peroxidase blocking solution (Dako Cytomation, Glostrup, Denmark). After several washes with KPBS, sections were incubated overnight at room temperature with the following polyclonal antibodies: (i) rabbit anti-tyrosine hydroxylase (TH) (Chemicon-Millipore, Temecula, CA, USA) used at 1/200; (ii) rat anti-mouse Cd68 antibody (AbD Serotec, Oxford, UK) used at 1/200; or (iii) rabbit anti-mouse GFAP antibody (Dako Cytomation, Glostrup, Denmark) used at 1/200. In the case of LAMP-1 immunostaining, we used the hybridoma monoclonal rat anti-mouse LAMP-1 antibody 1D4B, which was deposited by Dr. J. Thomas in the Developmental Studies Hybridoma Bank (DSHB; Hybridoma Product 1D4B), created by the NICHD (NIH, Bethesda, MD, USA) and maintained at The University of Iowa, Department of Biology, Iowa City, IA, USA. Dilutions were carried out in KPBS containing 2% bovine serum albumin and 0.1% Triton X-100 (Sigma Chem., Madrid, Spain). After incubation, sections were washed in KPBS, followed by incubation with the corresponding biotinylated secondary antibody (1/200) (Vector Laboratories, Burlingame, CA, USA) for 1 h at room temperature. Avidin-biotin complex (Vector Laboratories, Burlingame, CA, USA) and 3,3′-diaminobenzidine substrate–chromogen system (Dako Cytomation, Glostrup, Denmark) were used to obtain a visible reaction product. Negative control sections were obtained using the same protocol with omission of the primary antibody. A Leica DMRB microscope and a DFC300FX camera (Leica, Wetzlar, Germany) were used for the observation and photography of the slides, respectively. For quantification of TH, LAMP-1, GFAP, or Cd68 immunostaining in the substantia nigra, we used the NIH Image Processing and Analysis software (ImageJ; NIH, Bethesda, MD, USA) using 4–5 sections, separated approximately by 200 µm, and observed with 5x-20x objectives depending on the method and the brain area under quantification. In all sections, the same area of the substantia nigra pars compacta was analyzed. Analyses were always conducted by experimenters who were blinded to all animal characteristics. Data were expressed as a percentage of immunostaining intensity in the ipsilateral (lesioned) side over the contralateral (non-lesioned) side.

#### 4.4.3. Nissl Staining

Slices were used for Nissl staining using cresyl violet, as previously described [82], which permitted us to determine the effects of particular treatments on cell numbers. A Leica DMRB microscope (Leica, Wetzlar, Germany) and a DFC300Fx camera (Leica) were used to study and photograph the tissue, respectively. To count the number of Nissl-stained motor neurons (>400 μm^2^) in the ventral horn, high-resolution photomicrographs were taken with a 10× objective under the same conditions of light, brightness, and contrast. Counting was carried out with ImageJ software (U.S. National Institutes of Health, Bethesda, MD, USA, http://imagej.nih.gov/ij/, 1997–2012). At least 6 images per animal were analyzed to establish the mean of all animals studied in each group. Analyses were always conducted by experimenters who were blinded to all animal characteristics. In all analyses, data were transformed to the percentage over the mean obtained in the wild-type group for each parameter.

#### 4.4.4. Immunofluorescence Analysis in the ALS Experiment

Spinal slices were used for the detection and quantification of GFAP or Iba-1 immunofluorescence. After preincubation for 1 h with Tris-buffered saline with 0.1% Triton X-100 (pH 7.5), sections were sequentially incubated overnight at 4 °C with the following polyclonal antibodies: (i) anti-Iba-1 (Wako Chemicals, Richmond, VI, USA) used at 1:500; or (ii) anti-GFAP (Dako Cytomation, Glostrup, Denmark) used at 1:200, followed by washing in Tris-buffered saline and a new incubation (at 37 °C for 2 h) with an anti-rabbit secondary antibody conjugated with Alexa 488 (Invitrogen, Carlsbad, CA, USA). A DMRB microscope and a DFC300Fx camera (Leica, Wetzlar, Germany) were used for slide observation and photography. The mean density of immunolabelling was measured in the selected areas. Again, all data were transformed to the percentage over the mean obtained in the wild-type group for each parameter.

### 4.5. Real Time qRT-PCR Analysis

Tissues (striatum and spinal cord) from in vivo experiments and cell pellets from the in vitro experiments were also used for qRT-PCR analysis. Total RNA was isolated from the different samples using Trizol reagent (Sigma-Aldrich, Madrid, Spain). The total amount of RNA extracted was quantitated by spectrometry at 260 nm and its purity from the ratio between the absorbance values at 260 and 280 nm. After genomic DNA was removed (to eliminate DNA contamination), single-stranded complementary DNA was synthesized from up to 1 μg of total RNA using the commercial kits Rneasy Mini Quantitect Reverse Transcription (Qiagen, Hilgen, Germany) and iScript^TM^ cDNA Synthesis Kit (Bio-Rad, Hercules, CA, USA). The reaction mixture was kept frozen at −20 °C until enzymatic amplification. Quantitative RT-PCR assays were performed using TaqMan Gene Expression Assays (Applied Biosystems, Foster City, CA, USA) to quantify mRNA levels for TNF-α (ref. Mm99999068_m1), IL-1β (ref. Mm00434228_m1), iNOS (ref. Mm01309902_m1), COX-2 (ref. Mm00478372_m1), CB_1_ receptor (ref. Mm00432621_s1), CB_2_ receptor (ref. Mm00438286_m1), GPR55 (ref. Mm03978245_m1), and PPARγ (ref. Mm01184322_m1), using GAPDH expression (ref. Mm99999915_g1) as an endogenous control gene for normalization. The PCR assay was performed using the 7300 Fast Real-Time PCR System (Applied Biosystems, Foster City, CA, USA), and the threshold cycle (Ct) was calculated by the instrument’s software (7300 Fast System, Applied Biosystems, Foster City, CA, USA). Expression levels were calculated using the 2^−ΔΔCt^ method.

### 4.6. Statistics

Data were assessed using one-way or two-way (repeated measures) ANOVA, as required, followed by the Tukey test, or using the Student’s *t*-test, as required, using GraphPad Prism, version 8.00 for Windows (GraphPad Software, San Diego, CA, USA). A *p*-value lower than 0.05 was used as the limit for statistical significance. The sample sizes in the different experimental groups were always ≥ 5.

## 5. Conclusions

Therefore, our findings support the view that targeting the GPR55 with cannabinoids able to activate this receptor may afford neuroprotection in experimental PD, in particular, in models associated with mitochondrial dysfunction as in 6-OHDA-lesioned mice. Some beneficial effects were also found in LPS-lesioned mice, but with no effect against the intense glial activation occurring in this model. Future studies are projected to explore whether VCE-006.1 could also be active in mutant α-synuclein-based models of PD. Such a question is important to determine whether VCE-00.1 activity occurs exclusively in toxin-based models of PD or may also be found in models based on gene modifications. The need for this confirmation derives in part from the fact that VCE-006.1 was poorly active in experimental genetic models of ALS, although it is also possible that its development in this disease would require its combination with other cannabinoids active at additional endocannabinoid-related targets, in particular, anti-inflammatory targets. Collectively, these results demonstrate the specificities for the development of cannabinoid-based therapies for the different neurodegenerative disorders.

## Data Availability

Data supporting reported results may be supplied upon request to the authors.

## Sample Availability

Samples of the compounds are available from the authors.
